# Emergence of *Aeromonas veronii* strain co-harboring *bla*_KPC–2_, *mcr-3.17*, and *tmexC3.2-tmexD3.3-toprJ1b* cluster from hospital sewage in China

**DOI:** 10.3389/fmicb.2023.1115740

**Published:** 2023-05-17

**Authors:** Zhichen Zhu, Shuhua Wu, Jie Zhu, Tao Wang, Yicheng Wen, Chengcheng Yang, Jinnan Lv, Haifang Zhang, Liang Chen, Hong Du

**Affiliations:** ^1^Department of Clinical Laboratory, The Second Affiliated Hospital of Soochow University, Suzhou, Jiangsu, China; ^2^Department of Geriatrics, The Second Affiliated Hospital of Soochow University, Suzhou, Jiangsu, China; ^3^Department of General Practice, The Second Affiliated Hospital of Soochow University, Suzhou, Jiangsu, China; ^4^Hackensack Meridian Health Center for Discovery and Innovation, Nutley, NJ, United States; ^5^Hackensack Meridian School of Medicine, Seton Hall University, Nutley, NJ, United States

**Keywords:** *Aeromonas veronii*, *bla*_KPC–2_, *mcr-3.17*, *tmexCD3-toprJ1b*, hospital sewage

## Abstract

**Introduction:**

The raise of multi-drug resistant bacteria involving carbapenem, colistin, or tigecycline resistance constitutes a threat to public health, which partly results from the transmission of corresponding mobile resistance genes, such as *bla*_KPC_ and *bla*_NDM_ for carbapenem, *mcr* for colistin, and *tmexCD-toprJ* gene cluster for tigecycline. Herein, we described the emergence of an *Aeromonas veronii* strain HD6454 co-harboring *bla*_KPC–2_, *mcr-3.17*, and *tmexC3.2-tmexD3.3-toprJ1b* gene cluster from hospital sewage.

**Methods:**

Whole genome sequencing (WGS) was used to determine the genome sequence of HD6454, and the detailed genomic analysis of genetic elements or regions carrying key antimicrobial resistance genes (ARGs) from HD6454 were performed. Cloning experiment was conducted to confirm the function of key ARGs in mediating antimicrobial resistance. Conjugation experiment was conducted to determine the mobility of the plasmid.

**Results:**

The results showed that this strain belonged to a novel sequence type (ST) variant ST1016, and carried 18 important ARGs. Among them, the *bla*_KPC–2_ was carried by non-self-transmissible IncP-6 plasmid, while *tmexC3.2-tmexD3.3-toprJ1b* gene cluster and *mcr-3.17* were carried by integrative and mobilizable element (IME) or IME-related region in chromosome. The *mcr-3.17*, *mcr-3.6*, and *mcr-3-like3* genes were further inferred to originate from IMEs of *Aeromonas* species. Additionally, for the first time, the *mcr-3.17* was confirmed to confer low-level resistance to colistin under inducible expression, while *tmexC3.2-tmexD3.3-toprJ1b* gene cluster was confirmed to confer low-level resistance to tigecycline.

**Discussion:**

This is the first report of a strain co-harboring *bla*_KPC–2_, *mcr-3.17*, and *tmexC3.2-tmexD3.3-toprJ1b* gene cluster. Although the resistance and/or mobility of these ARGs are limited in this strain, the emergence of this multiple important ARGs-carrying strain deserves further attention.

## Introduction

The rising bacterial resistance to carbapenems is a worldwide threat to public health. Carbapenem resistance is mainly caused by the expression of carbapenemase-encoding genes (*bla*_KPC_, *bla*_NDM_, *bla*_VIM_, etc.) (Nordmann and Poirel, 2019). Meanwhile, colistin and tigecycline are considered as the last-resort for the treatment of life-threatening infections caused by multi-drug resistant Gram-negative bacteria, especially the carbapenem-resistant strains (Doi, 2019; Gonzalez-Avila et al., 2021). However, global emergence of colistin/tigecycline-resistant pathogens has been increasingly reported (El-Sayed Ahmed et al., 2020; Yaghoubi et al., 2021), which partly results from the transmission of mobile resistance genes, including *mcr* for colistin (Anyanwu et al., 2020), and *tet*(X) (Fang et al., 2020) and resistance-nodulation-division (RND) efflux pump gene cluster *tmexCD-toprJ* (Lv et al., 2020; Wang et al., 2021b,c) for tigecycline. Co-carriers of carbapenem-resistant and colistin-resistance genes [such as *bla*_KPC_ and *mcr-3.3* (Tang et al., 2020)], or carbapenem-resistant and tigecycline-resistance genes [such as *bla*_NDM–4_, *tet*(X), and *tmexCD3-toprJ3* (Hirabayashi et al., 2021)] in bacteria have been reported. These co-carriers of antibiotic resistance genes (ARGs) reduce the options of clinical antibiotic treatment, which raise a significant concern.

*Aeromonas* species are Gram-negative bacteria, and mainly infect aquatic organisms (Fernández-Bravo and Figueras, 2020). *Aeromonas* species can cause intestinal and extra-intestinal infections in human (Gowda et al., 2015). Although *Aeromonas* species widely distribute in diverse ecosystems, they are more commonly isolated from water, such as drinking water, seawater and hospital sewage (Fernández-Bravo and Figueras, 2020). Among them, hospital sewage plays an important role in the spread of ARGs in *Aeromonas* (Karkman et al., 2018; Zhang et al., 2020).

The potential association between *Aeromonas* species and carbapenem/tigecycline/colistin genes has been reported. Firstly, Nwaiwu and Aduba (2020) discovered that *bla*_KPC–2_ gene was the most carbapenemase-encoding gene carried by *Aeromonas* species plasmids within GenBank (9.52%, 10/105). Secondly, *tmexCD*-*toprJ*-carrying *Aeromonas* strains have also been gradually reported (Dong et al., 2022; Wu et al., 2023). Thirdly, since the first mobile colistin resistance (*mcr*) gene *mcr-1* was characterized in Enterobacteriaceae in 2016 (Liu et al., 2016), additional nine mobile colistin resistance genes (*mcr-2*∼*mcr-10*) and various variants have been reported (El-Sayed Ahmed et al., 2020; Wang et al., 2020a). Among them, *mcr-3* is considered most likely to originate from *Aeromonas* species (Yin et al., 2017; Shen et al., 2018). Further investigation could help clarify the mechanism of transmission of *mcr-3* in *Aeromonas* species. Additionally, most *mcr-3* variants have only been characterized by sequence analysis, their contributions to colistin resistance are not described.

In this study, we describe the emergence of an *Aeromonas veronii* strain co-harboring *bla*_KPC–2_, *mcr-3.17*, and *tmexC3.2-tmexD3.3-toprJ1b* gene cluster from hospital sewage for the first time. A detailed genomic dissection analysis was conducted to decipher the genomic characteristics of this strain.

## Materials and methods

### Bacterial strains

The *A. veronii* strain, HD6454, was isolated from the raw sewage in sewage conduit under washbasin in digestive department of the Second Affiliated Hospital of Soochow University (Suzhou, China) in July 2019. The detailed isolation methodology was described previously (Wen et al., 2022). Bacterial species identification was carried out using Matrix-Assisted Laser Desorption/Ionization Time of Flight Mass Spectrometry (MALDI-TOF-MS). The presence of carbapenemase genes (*bla*_KPC_, *bla*_NDM_, *bla*_VIM_, *bla*_OXA–48–like_, and *bla*_IMP_), *mcr* genes and *tmexCD-toprJ* was determined by PCR amplification as described previously (Lv et al., 2020; Tang et al., 2020).

### Sequencing and sequence assembly

Bacterial genomic DNA was isolated using the Omega Bio-Tek Bacterial DNA Kit (Doraville, GA, USA), and sequenced from a sheared DNA library with average size of 10 kb on a Nanopore PromethION platform (Oxford Nanopore Technologies, OX, UK), as well as a paired-end library with an average insert size of 350 bp on a NovaSeq sequencer (Illumina, CA, USA). A hybrid assembly was then conducted by *Unicycler* 0.4.9^1^ using both paired-end short Illumina reads and the long Nanopore reads.

### Multi-locus sequence typing (MLST) analysis and phylogenetic analysis

The sequence type (ST) of the *A. veronii* strain HD6454 was identified according to the online multi-locus sequence typing (MLST) scheme.^2^ Amino acid sequences of MCR variants and MCR-3-like variants were aligned using *ClustalW* in *MEGAX* 10.1.8 (Kumar et al., 2018). Unrooted maximum-likelihood phylogenetic trees were further generated using *MEGAX* 10.1.8 with a bootstrap iteration of 1,000.

### Sequence annotation and comparison

Open reading frames (ORFs) and pseudogenes were predicted using *RAST* 2.0 (Brettin et al., 2015) combined with *BLASTP/BLASTN* searches (Boratyn et al., 2013) against the *UniProtKB/Swiss-Prot* database (Boutet et al., 2016) and the *RefSeq* database (O’Leary et al., 2016). Annotation of resistance genes, mobile elements, and other features were carried out using the online databases including *CARD* (Jia et al., 2017), *ResFinder* 4.1 (Zankari et al., 2012), *Danmel* (Wang et al., 2022), *ISfinder* (Siguier et al., 2006), and *Tn Number Registry* (Tansirichaiya et al., 2019). Multiple and pairwise sequence comparisons were performed using *BLASTN*. Gene organization diagrams were drawn through scripts from *Danmel* (Wang et al., 2022), and displayed using *Inkscape* 1.0.^3^ Prediction of protein secondary structure was performed using *ESPript* 3.0 (Robert and Gouet, 2014) and displayed using *Phyre* 2.0 (Kelley et al., 2015).

### Cloning experiments

The construct of recombinant plasmid was performed through seamless cloning by ClonExpress Ultra One Step Cloning Kit (Vazyme, China). The details were described in Supplementary text, and the primers used herein were shown in Supplementary Table 1. The resulting recombinant plasmids were transformed through heat shock into *Escherichia coli* DH5α, and 100 μg/ml ampicillin was used for the transformant selection. The transformants DH5α/pUC18, DH5α/pBAD24 and DH5α/pBAD24-*mcr*-*1.1* were also constructed as controls. Successful transformants were confirmed by PCR, and Sanger sequencing on a 3730XL sequencer (ABI, Boston, MA, USA). Induction of the pBAD24 vector was performed using MH II broth (Cation-Adjusted) supplemented with 0.4% L-arabinose as previously described (Kieffer et al., 2019).

### Bacterial antimicrobial susceptibility test

The susceptibility of colistin and tigecycline was carried by the minimum inhibitory concentrations (MICs) method. Results were interpreted according to the European Committee on Antimicrobial Susceptibility Testing (EUCAST).^4^ The susceptibility of other antimicrobial agents (Supplementary Table 2) was carried by using the Phoenix System-M50 automatic microbiology analyzer (BD, USA). Results were interpreted according to the 2020 Clinical and Laboratory Standards Institute (CLSI) guidelines. The susceptibility test through MIC method was repeated three times to ensure the accuracy of the result. The *E. coli* ATCC 25922 was used as the quality control.

### Conjugal transfer

Conjugal transfer experiment was carried out with sodium azide-resistant *E. coli* J53 as the recipient, and the HD6454 isolate as the donor. In brief, overnight cultures of the HD6454 and J53 were mixed at a ratio of 1:1 in LB broth, and the mixture was then spotted on a hydrophilic nylon membrane filter with a 0.45 μm pore size (Millipore) that was placed on LB agar plate and then incubated for mating at 37 C for 18 h. Bacteria were washed from filter membrane and spotted on LB agar plate, and 200 μg/ml sodium azide (for J53) together with 2 μg/ml imipenem (for *bla*_KPC_) was used for selecting an *E. coli* transconjugant carrying *bla*_KPC_.

### Nucleotide sequence accession numbers

The complete chromosome and plasmid sequences of HD6454 were submitted to GenBank under accession numbers CP079823-CP079826, respectively.

## Results

### Characteristics of HD6454

The *A. veronii* strain HD6454 was isolated from the raw sewage in sewage conduit under washbasin of digestive department, and belonged to a novel ST variant ST1016. The strain harbored a 5,029,035-bp chromosome genome and three plasmids: pHD6454-KPC, pHD6454-2, and pHD6454-3 (Table 1). Meanwhile, HD6454 carried 18 important ARGs, including *bla*_KPC–2_, *mcr-3.17*, and *tmexC3.2-tmexD3.3-toprJ1b* gene cluster. Additionally, a *mcr-3-like* gene was found downstream of the *mcr-3.17* gene. This *mcr-3-like* gene was further designated as *mcr-3-like3* as previously described (Shen et al., 2018). Susceptibility testing results showed that HD6454 was multidrug-resistant, including carbapenems (Supplementary Table 2). However, despite carrying *mcr-3.17* and *tmexC3.2-tmexD3.3-toprJ1b* gene cluster, HD6454 was susceptible to colistin (2 mg/L) and tigecycline (0.125 mg/L) based on the EUCAST clinical breakpoints (see footnote 4).

**TABLE 1:** Antimicrobial resistance genes and plasmids carried by HD6454.

Characteristic	Chromosome	Plasmids		
		pHD6454-KPC	pHD6454-2	pHD6454-3
Size (bp)	5,029,035	51,662	13,893	10,228
Replicon type	–	IncP-6 (IncG)	Unknown	Unknown
Accession number	CP079823	CP079826	CP079824	CP079825
Resistance gene(s)	*mcr-3.17*, *tmexC3.2-tmexD3.3-toprJ1b*, *aacA3*, *aacA4*, *aadA16*, *strA*, *strB*, *bla*_OXA–21_, *cphA*, *qnrVC6*, *mph*(A), *mph*(E), *msr*(E), *catB3*, *arr-3*, *sul1*, *dfrA27*	*bla*_KPC–2_	–	–

### Functional identification of *mcr-3.17* and *mcr-3-like3*

The MCR-3.17 shared 89.24% amino acid sequence similarity to MCR-3.1 (accession number KY924928), while MCR-3-like3 shared 84.29% amino acid sequence similarity to MCR-3.1. The amino acid sequence of MCR-3.17 shared the highest similarity (93.15%) with MCR-3.6 (accession number MF598076) among all MCR-3 variants. The phylogenetic analysis also showed that, compared with other MCR-3 variants, the evolution relationship between MCR-3.6 and MCR-3.17 was the closest (Supplementary Figure 1). Nevertheless, MCR-3.17 was still different from MCR-3.6 in predicted protein secondary structure (Supplementary Figure 2). Previous studies showed that MCR proteins were predicted to have two conservative domains, the membrane-anchored domain (approximate residues range for MCR-3 was 1–172) and the soluble catalytic domain (approximate residues range for MCR-3 was 173 to the last) (Yin et al., 2017; Carroll et al., 2019). Compared with MCR-3.6, 33 mutations in amino acid sequences of the membrane-anchored domain of MCR-3.17 were identified, and further led the loss of two β-sheet areas in protein secondary structure, while MCR-3.17 also had four mutations in amino acid sequences of the soluble catalytic domain, and further led the increase of a 3_10_-helix area in protein secondary structure (Supplementary Figure 2).

In order to determine the function of *mcr-3.17* and *mcr-3-like3* in mediating colistin resistance, the transformants DH5α/pUC18-*mcr-3.17*, DH5α/pUC18-*mcr*-*3-like3* and DH5α/pUC18-*mcr-3.17*&*mcr*-*3-like3* were obtained. However, MIC of these three transformants displayed no difference, compared with MIC of DH5α/pUC18 (Table 2). Considering probable variations in the promoter activities of *mcr-3.17* and *mcr-3-like3* in HD6545, the transformants DH5α/pBAD24-*mcr-3.17* and DH5α/pBAD24-*mcr-3-like3* were further constructed. In addition, transformants DH5α/pBAD24 and DH5α/pBAD24-mcr-1.1 were also constructed as controls. The antimicrobial susceptibility testing showed that DH5α/pBAD24-*mcr*-*3.17* presented an MIC of 4 mg/L after induction of arabinose, while DH5α/pBAD24-*mcr*-1.1 had an MIC of 16 mg/L. On the contrary, DH5α/pBAD24-*mcr*-*3-like3* presented a MIC of 1 mg/L after induction of arabinose (Table 2). This result suggested that *mcr*-*3.17*, but not *mcr-3-like3*, could confer low-level resistance to colistin.

**TABLE 2:** Antimicrobial drug susceptibility profile.

Bacterial isolate	Minimum inhibitory concentration (mg/L)/antimicrobial susceptibility		
	Colistin^a^	Colistin^b^	Tigecycline
DH5α/pUC18-*mcr-3.17*	2/S*	–	–
DH5α/pUC18-*mcr-3-like3*	2/S	–	–
DH5α/pUC18-*mcr-3.17*&*3-like3*	2/S	–	–
DH5α/pUC18	2/S	–	–
DH5α/pBAD24-*mcr-3.17*	2/S	4/R	–
DH5α/pBAD24-*mcr-3-like3*	2/S	1/S	–
DH5α/pBAD24-*mcr-1.1*	2/S	16/R	–
DH5α/pBAD24	2/S	2/S	–
DH5α/pUC18-*tmexC3.2-tmexD3.3-toprJ1b*	–	–	0.5/S
DH5α/pUC18	–	–	0.125/S
ATCC 25922	1/S	–	0.06/S

*S, sensitive; R, resistant.

^a^Non-induced with arabinose.

^b^Induced with arabinose.

### Genetic characterization of *mcr-3.17* and *mcr-3-like3*

**FIGURE 1:**
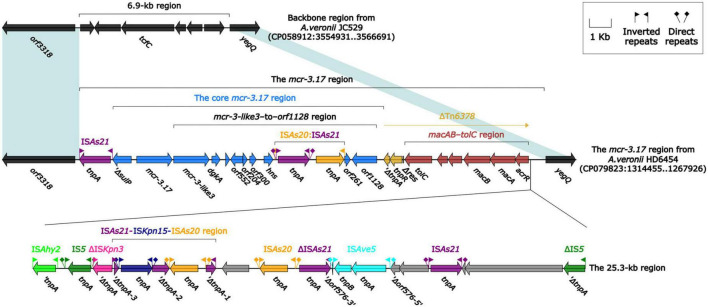
Organization of the *mcr-3.17* region from HD6454, and comparison to related region. Genes are denoted by arrows. Genes, mobile genetic elements and other features are colored based on their functional classification. Shading denotes regions of homology (nucleotide identity ≥ 95%).

The *mcr-3.17* and *mcr-3-like3* genes were located in a 46.5-kb *mcr-3.17* region in the chromosome of HD6454 (Figure 1). The *mcr-3-like3* gene was located immediately downstream of *mcr-3.17*, the nucleoside bases between them was 66 bp. Compared with the chromosome of a reference *A. veronii* strain JC529 (accession number CP058912), a 6.9-kb backbone region was replaced by the *mcr-3.17* region in HD6454. This *mcr-3.17* region contained a truncated Tn*6378*, a 25.3-kb region with multiple IS elements, and the “core *mcr-3.17* region,” including truncated *sulP* gene, *mcr-3.17* gene and *mcr-3-like3*–to–*orf1128* region.

**FIGURE 2:**
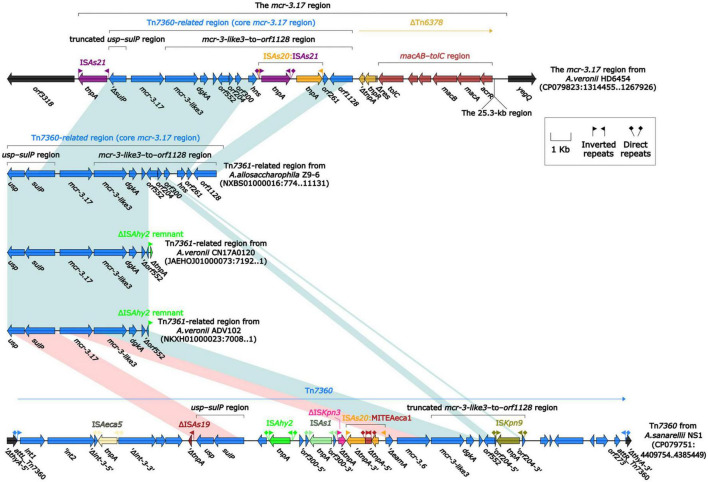
Organization of Tn*7360*-related regions, and comparison to Tn*7360*. The detailed information of strains is described in Supplementary Tables 3, 4. Genes are denoted by arrows. Genes, mobile genetic elements and other features are colored based on their functional classification. Shading denotes regions of homology (light blue: ≥95% nucleotide identity; light red: 88–95% nucleotide identity).

**FIGURE 3:**
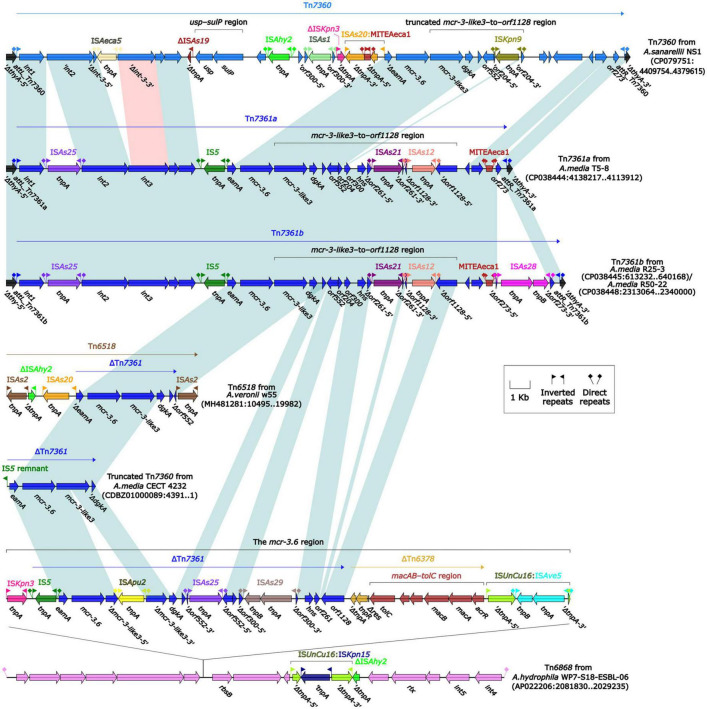
Organization of *mcr-3.6-*carrying genetic elements or related regions. The detailed information of strains is described in Supplementary Tables 3, 4. Genes are denoted by arrows. Genes, mobile genetic elements and other features are colored based on their functional classification. Shading denotes regions of homology (light blue: ≥95% nucleotide identity; light red: 88–95% nucleotide identity).

To further probe how this 46.5-kb *mcr-3.17* region originated, a detailed genetic dissection analysis was applied to compare the genetic structure of HD6454 with additional 10 *mcr-3.6* (*n* = 7) or *mcr-3.17* (*n* = 3)-harboring genomes download form the GenBank (Supplementary Tables 3, 4). Similar core *mcr-3.17* regions could be found in three *mcr-3.17-*carried contig fragments from *Aeromonas allosaccharophila* Z9-6 (Shen et al., 2018), and *A. veronii* CN17A0120 and ADV102 (Rangel et al., 2019; Figure 2). Truncated or complete *usp*–*sulP* region and truncated or complete *mcr-3-like3*–to–*orf1128* regions could be found upstream and downstream of the *mcr-3.17* gene from all *mcr-3.17* regions in this study, respectively. However, due to the limited length of these contig assemblies, whether these core *mcr-3.17* regions were carried by complete genetic elements or not could not be determined. Fortunately, an integrative and mobilizable element (IME) from *A. sanarellii* NS1 was found to share at least 96.71% identity with these core *mcr-3.17* regions (Figure 2). This IME harbored *mcr-3.6* and *mcr-3-like3* genes, and was newly designated Tn*7360*. Similar *usp*–*sulP* region and truncated *mcr-3-like3*–to–*orf1128* region could also be found in Tn*7360*. Therefore, the so-called core *mcr-3.17* region was further named as Tn*7360*-related region. Moreover, except Tn*7360*, all *mcr-3.6* and *mcr-3-like3* genes were carried by IME Tn*7361a/b* (Shi et al., 2020) or related genetic elements and regions (Figure 3). The backbone sequence of Tn*7361a/b* was 95.60% identical to the backbone sequence of Tn*7360* with 76% coverage. Tn*7361a/b* also harbored the complete *mcr-3-like3*–to–*orf1128* region. Meanwhile, Tn*7360* and Tn*7361a/b* were all integrated within the *thyA* gene with 6-bp direct repeats (DRs). In addition, truncated Tn*7360* fragments were identified in other genetic elements, including composite transposon Tn*6518* (Wang et al., 2020b) from *A. veronii* w55 and IME Tn*6868* from *A. hydrophila* WP7-S18-ESBL-06.

In summary, all *mcr-3.6*, *mcr-3.17* and *mcr-3-like3* characterized in this study were located within IME Tn*7360/*Tn*7361* or related genetic elements and regions from *Aeromonas* species.

### Functional identification of *tmexC3.2-tmexD3.3-toprJ1b* gene cluster

A *tnfxB3-tmexCD3-toprJ1*-like gene cluster was found in the chromosome of strain HD6454 initially. The sequence of this cluster was of 100% identical to the one carried by chromosome of *A. caviae* WCW1-2 (accession number CP039832) (Wang et al., 2021b). Furthermore, according to the naming scheme previously described (Wang et al., 2021a): (1) compared with the *tnfxB3* gene from *Proteus cibarius* strain SDQ8C180-2T (accession number CP073356), one mutation was identified in the *tnfxB3-like* gene from HD6454 (predicted to encode Thr46Ala), and this *tnfxB3-like* gene was thus named *tnfxB*3.2; (2) compared with the *tmexD3* gene from strain SDQ8C180-2T, one mutation was identified in the *tmexD3-like* gene from HD6454 (predicted to encode Val56Glu). This *tmexD3-like* gene was also different from the *tmexD3.2* gene in strain *P. mirabilis* SDY9C89-2 (accession number MZ004963), so it was named *tmexD3.3*; (3) the *tmexC3-like* gene and *toprJ1-like* gene from HD6454 were identical to the *tmexC3.2* gene from *Pseudomonas aeruginosa* strain AHM8C91AI (accession number JAGSOC000000000) and the *toprJ1b* gene from strain SDQ8C180-2T, respectively. In general, this *tnfxB3-tmexCD3-toprJ1*-like gene cluster from HD6454 was identified as *tnfxB3.2-tmexC3.2-tmexD3.3-toprJ1b* finally.

In order to determine the function of *tmexC3.2-tmexD3.3-toprJ1b* in mediating tigecycline resistance, the transformants DH5α/pUC18-*tmexC3.2-tmexD3.3-toprJ1b* was obtained. The antimicrobial susceptibility testing showed that DH5α/pUC18-*tmexC3.2-tmexD3.3-toprJ1b* presented an MIC of 0.5 mg/L to tigecycline, while DH5α/pUC18 had an MIC of 0.125 mg/L (Table 2). This result suggested that *tmexC3.2-tmexD3.3-toprJ1b* could confer low-level resistance to tigecycline.

### Genetic characterization of *tmexC3.2-tmexD3.3-toprJ1b* gene cluster

**FIGURE 4:**
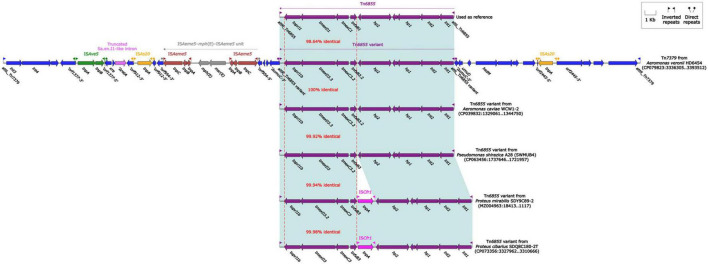
Organization of Tn*7379* from HD6454, and comparison to related Tn*6855* and its variants. Genes are denoted by arrows. Genes, mobile genetic elements and other features are colored based on their functional classification. Shading denotes regions of homology (light blue: ≥98% nucleotide identity). The accession number of Tn*6855* (Yu et al., 2021b) used as reference is MK347425.

The *tmexCD1-toprJ1* gene cluster was firstly identified in the structure “*int1-int2-hp1-hp2-tnfxB1-tmexCD1-toprJ1*,” and this structure was further carried by Tn*5393* (Lv et al., 2020). Subsequently, this structure along with its attachment site at the left/right end (*attL/R*) was defined as relaxosome-missing IME Tn*6855* (Yu et al., 2021b). Further genetic dissection analysis showed that the *tnfxB3.2-tmexC3.2-tmexD3.3-toprJ1b* gene cluster from HD6454 was located within an IME Tn*6855* variant, which was integrated in the *umuC* gene of the novel IME Tn*7379* with 6-bp DRs (Figure 4). This Tn*6855* variant differed from Tn*6855* by the substitution of *tnfxB1-tmexCD1-toprJ1* gene cluster to *tnfxB3.2-tmexC3.2-tmexD3.3-toprJ1b.* Additionally, some other *tnfxB3-tmexCD3-toprJ1b* gene clusters were also located within similar Tn*6855* variants (Figure 4), and integrated within the *umuC* genes as previously reported (Wang et al., 2021a,b).

### The *bla*_KPC–2_-carrying IncP-6 plasmid pHD6454-KPC

**FIGURE 5:**
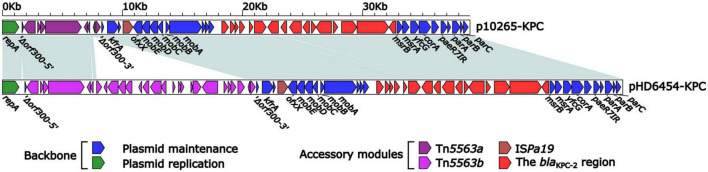
Linear comparison of IncP-6 plasmids pHD6454-KPC and p10265-KPC. Genes are denoted by arrows. Genes, mobile genetic elements and other features are colored based on function classification. Shading regions denote homology of two plasmids (light blue: ≥99% nucleotide identity).

**FIGURE 6:**
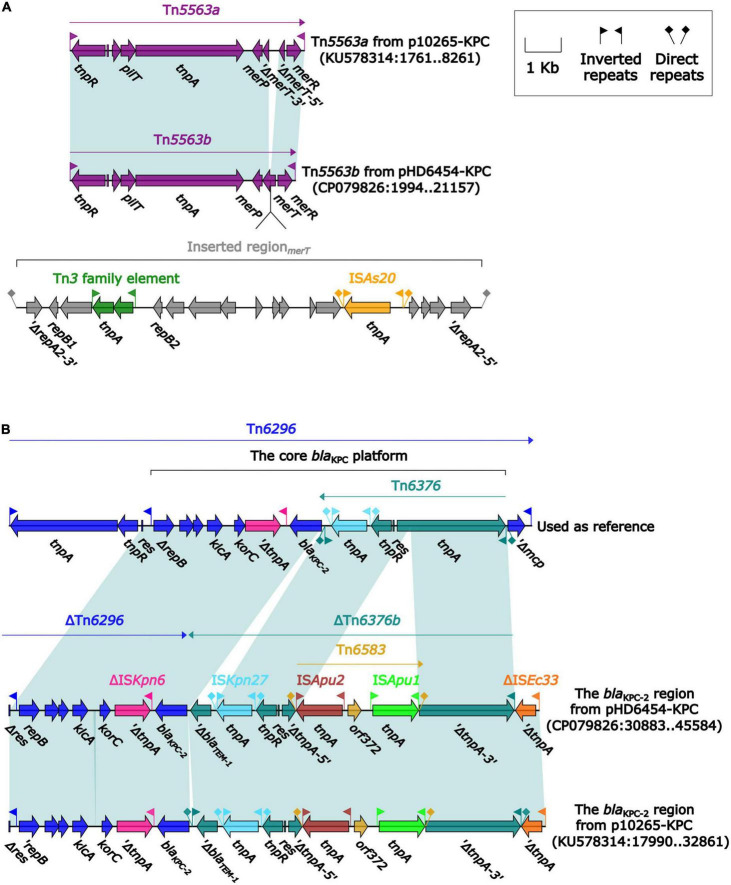
Organization of **(A)** the *bla*_KPC–2_ region and **(B)** Tn*5563b* from pHD6454-KPC, and comparison to related genetic elements. Genes are denoted by arrows. Genes, mobile genetic elements and other features are colored based on their functional classification. Shading denotes regions of homology (light blue: ≥ 99% nucleotide identity). The accession number of Tn*6296* (Wang et al., 2017) used as reference are FJ628167.

The *bla*_KPC–2_ was carried by a 51.66-kb IncP-6 plasmid, which was assigned the name pHD6454-KPC. The modular structure of pHD6454-KPC was divided into the backbone and three accessory modules (IS*Pa19*, Tn*5563b* and the *bla*_KPC–2_ region) which were resulted from exogenous DNA regions insertion at different sites of the backbone (Supplementary Figure 3). The plasmid pHD6454-KPC shared 99.99% nucleotide identity to the IncP-6 reference plasmid p10265-KPC from *Pseudomonas aeruginosa* 10,265 with 76% coverage (accession number KU578314) (Dai et al., 2016), while the backbone of them were 99.98% nucleotide identical, along with 99% coverage. This showed that these two plasmids were mainly different in accessory modules (Figure 5). The unit transposon Tn*5563b* in pHD6454-KPC differed from Tn*5563a* in p10265-KPC by the insertion of a *merT*-harboring region (inserted region*_merT_*, 12.90 kb) (Figure 6A). The inserted region*_merT_* was flanked by 9-bp DRs, and may originated from the transposition and homologous recombination of an unknown-type plasmid. Additionally, the *bla*_KPC–2_ region in pHD6454-KPC was composed of a truncated *bla*_KPC–2_-carrying unit transposon Tn*6296* (Wang et al., 2017) and a truncated unit transposon Tn*6376b*, and was almost identical to the one in p10265-KPC (Figure 6B).

The genomic dissection analysis showed that pHD6454-KPC lost the *tra* module, which was consistent with other IncP-6 plasmids as described previously (Dai et al., 2016; Hu et al., 2019). Repeated conjugation attempts failed to transfer pHD6454-KPC from strain HD6454 into J53, which matched the sequence analysis result.

## Discussion

The *mcr-3* genes have spread widely into diverse environmental niches by horizontal and vertical transfer (Anyanwu et al., 2020). Since the *mcr-3.1* gene was initially identified in China in 2017, at least 40 non-redundant *mcr-3* variants have been reported in Asia, North America, Africa, and Europe (El-Sayed Ahmed et al., 2020; Snyman et al., 2021; Stosic et al., 2021; Yu et al., 2021a). Among them, *mcr-3.17* was only reported once in 2018, which was identified in *A. allosaccharophila* isolated from chicken meat in China (Shen et al., 2018). However, neither the detailed genomic structure of *mcr-3.17* nor the resistant phenotype of *mcr-3.17* to colistin have been confirmed in the previous study. In this study, *mcr-3.17* was found in the hospital environment, and was confirmed to confer low-level resistance to colistin only under inducible expression. The *mcr-3.6* gene has been proved to confer high-level resistance to colistin (Wang et al., 2020b). Although *mcr-3.6* gene shares the highest similarity with *mcr-3.17* among all *mcr-3* variants, they still show several differences in both amino acid sequence and protein secondary structure, which may explain the low-level resistance to colistin of *mcr-3.17*. However, the key mutations affecting the level of resistance of *mcr-3* variants to colistin remain unknown and need to be further investigated. As for *mcr-3-like* gene, a total of four *mcr-3-like* variants have been reported (Ling et al., 2017; Shen et al., 2018), which are all downstream of *mcr-3* variants. Moreover, the nucleoside bases between these *mcr-3-like* variants and the corresponding *mcr-3* variants upstream of them are always 66 bp. Previous studies have preliminarily proved that *mcr-3-like1* and *mcr-3-like3* could not mediate the MIC changes in recipient strains (Ling et al., 2017; Wang et al., 2020b). In this study, whether *mcr-3-like3* gene was cloned into the cloning vector pUC18 together with its upstream promoter-proximal region or induced in the expression vector pBAD24, it could also not mediate the resistance to colistin. This result further proves that the MCR-3-like3 has no resistance activity to colistin.

Several *tmexCD3-toprJ1b* variants have been identified in various species, including *Aeromonas* spp. (Hirabayashi et al., 2021; Wang et al., 2020b,2021a,c; Wu et al., 2023). Although the identical sequence has been reported, the *tmexC3.2-tmexD3.3-toprJ1b* gene cluster was named systematically and was confirmed to confer low-level resistance to tigecycline for the first time in this study.

It was noteworthy that, despite carrying *mcr-3.17* and *tmexC3.2-tmexD3.3-toprJ1b* gene cluster, HD6454 was susceptible to colistin and tigecycline. Since *mcr-3.17* could confer low-level resistance to colistin only under inducible expression, namely, the high expression level of *mcr-3.17*, the phenomenon that HD6454 was susceptible to colistin might result from the low expression level of *mcr-3.17* in HD6454. Meanwhile, *tnfxB3* gene has been confirmed to have the transcriptional repression function to downstream *tmexCD3-toprJ1b* (Wang et al., 2021a). Hence, the transcriptional repression function of *tnfxB3.2* may explain the phenomenon that HD6454 was susceptible to tigecycline, which deserves further study.

Integrative and mobilizable elements are not self-transmissible, and their intercellular mobility is achieved with utilization of the conjugation machinery of unrelated co-resident conjugative element (Guédon et al., 2017). In this study, both *tnfxB3.2-tmexC3.2-tmexD3.3-toprJ1b* gene cluster and *mcr-3.17* were identified to be associated with IMEs. Among them, *tnfxB3.2-tmexC3.2-tmexD3.3-toprJ1b* gene cluster was carried by Tn*6855* variant. Such Tn*6855* variants could further integrate into the *umuC* gene of various genetical elements, for example, SXT/R391 family integrative and conjugative elements (Wang et al., 2021a,c), a IncC-IncX3 hybrid plasmid pNUITM-VK5_mdr (Hirabayashi et al., 2021), and another IME Tn*7379* identified herein. Meanwhile, all *mcr-3.6*, *mcr-3.17* and *mcr-3-like3* characterized in this study were located within IME Tn*7360/*Tn*7361* or related genetic elements [such as composite transposon Tn*6518* (Wang et al., 2020b) and IME Tn*6868*] and regions. This shows that IME serves as an important carrier and mediator in the transmission of *tnfxB3-tmexCD3-toprJ1b* gene cluster and partial *mcr-3* variants.

Due to the lack of a *tra* module encoding primary pilus, IncP-6 plasmid was previously considered to be not self-transmissible (Dai et al., 2016; Hu et al., 2019). However, IncP-6 plasmid harbors conserved *par-rep* regions for partition-replication and *mob* gene module for mobilization, which allows it to transfer if the right self-transmissible plasmids are co-resident (Dai et al., 2016). This explains why the same *bla*_KPC–2_ encoding IncP-6 plasmid could be found in non-clonal different isolates within various species from clinical or environmental sources (Yao et al., 2017). Similarly, the backbone sequence of the *bla*_KPC–2_-carrying pHD6454-KPC found in this study was almost identical to the IncP-6-type plasmid p10265-KPC firstly reported in China (Dai et al., 2016). Moreover, the accessory modules of pHD6454-KPC also seemed to evolved from the ones in p10265-KPC (Dai et al., 2016) undergoing the events of gene acquisition and deletion. Therefore, the potential transmission of these *bla*_KPC–2_-carrying IncP-6 plasmids in China should be closely monitored.

To the best of our knowledge, this is the first report of a strain co-harboring *bla*_KPC–2_, *mcr-3.17*, and *tmexC3.2-tmexD3.3-toprJ1b* gene cluster. Several factors may contribute to the emergence of this *A. veronii* strain HD6454. Firstly, *mcr-3* is considered that most likely originated from *Aeromonas* species (Yin et al., 2017; Shen et al., 2018). All the 11 strains carrying *mcr-3.6* or *mcr-3.17* characterized in this study are *Aeromonas* species. Secondly, acquisition of exogenous DNA is a general property of *Aeromonas* environmental isolates (Huddleston et al., 2013), while the potential transmission of *mcr-3*-carrying or *tmexCD3-toprJ1b*-carrying IMEs (Guédon et al., 2017), and *bla*_KPC–2_-carrying IncP-6 plasmid increases the possibility of obtaining exogenous DNA by *Aeromonas* isolates. Thirdly, these ARGs and related genetic elements seem to be mainly distributed in China. Among them, *bla*_KPC–2_-bearing IncP-6 plasmid has been reported to be the most detected in China (Hu et al., 2019). Eight of the 11 strains carrying *mcr-3.6* or *mcr-3.17* characterized in this study are isolated from clinical or environmental sources in China. Furthermore, the current reports of *tmexCD3-toprJ1b* gene cluster are mainly based on strains from China (Wang et al., 2020b,2021a,c). These factors also show that *Aeromonas* species may act as important vectors for the dissemination of ARGs in China.

## Conclusion

In conclusion, this study identified the *A. veronii* strain HD6454 co-harboring *bla*_KPC–2_, *mcr-3.17*, and *tmexC3.2-tmexD3.3-toprJ1b* gene cluster for the first time. The *bla*_KPC–2_ was carried by IncP-6 plasmid, while *tmexC3.2-tmexD3.3-toprJ1b* gene cluster and *mcr-3.17* were carried by IME or IME-related region in chromosome. Although the resistance and/or mobility of these ARGs are limited in HD6454, the emergence of this multiple important ARGs-carrying strain deserves further attention.

## Data availability statement

The datasets presented in this study can be found in online repositories. The names of the repository/repositories and accession number(s) can be found in the article/Supplementary material.

## Author contributions

HD, LC, and ZZ conceived and designed the experiments. ZZ, JZ, and TW performed the experiments. ZZ, YW, and CY analyzed the genome sequence. ZZ and SW wrote the manuscript. HZ, SW, JL, YW, LC, and HD contributed other analysis or discussion. All authors contributed to the article and approved the submitted version.

## Footnotes

1
https://github.com/rrwick/Unicycler

2
https://pubmlst.org/

3
https://inkscape.org/en/

4
https://www.eucast.org/

## Supplementary material

Supplementary Figure 1 Evolution relationships of the 54 MCR variants and MCR-3-like variants. Degree of support (percentage) for each cluster of associated taxa, as determined by bootstrap analysis, is shown next to each branch. Bar corresponds to scale of sequence divergence.

Supplementary Figure 2 Comparison of amino acid sequences and protein secondary structures of MCR-3.6 and MCR-3.17. Within the amino acid sequence alignment, a strict identity was denoted by a red box with a white character. A yellow box around an amino acid residue denoted similarity across groups, while a residue in boldface denoted similarity within a group. Letter α, β, and η represented α-helix, β-sheet and 3_10_-helix, respectively. Green digits below the alignment denote cysteine residues forming a disulfide bridge.

Supplementary Figure 3 Schematic diagram of the plasmid pHD6454-KPC. Genes of different functions are denoted by arrows and presented in various colors. The circles show (from outside to inside): predicted coding sequences, scale in 10 kb, backbone (black) and accessory module (gray) regions, GC content and GC skew [(G–C)/(G + C)].
