# Supplementary material for: Emergence of Aeromonas veronii strain co-harboring blaKPC–2, mcr-3.17, and tmexC3.2-tmexD3.3-toprJ1b cluster from hospital sewage in China
Source: Front Microbiol. 2023 May 17;14:1115740. doi: 10.3389/fmicb.2023.1115740 (PMC10229833; doi:10.3389/fmicb.2023.1115740)
Supplement: Supplementary file 1 [file Data_Sheet_1.PDF]

The *mcr-3.17* coding region together with its immediately 402-bp upstream promoter-proximal region and 58-bp downstream terminator-proximal region, the *mcr-3-like3* coding region together with its immediately 180-bp upstream promoter-proximal region and 168-bp downstream terminator-proximal region, and the large fragment containing the above two regions from strain HD6454 were amplified through PCR and cloned into the ampicillin-resistant vector pUC18 (handled with XbaI enzyme) through seamless cloning by ClonExpress Ultra One Step Cloning Kit (Vazyme, China), respectively. The *mcr-1.1*, *mcr-3.17* and *mcr-3-like3* coding regions from strain HD6454 were amplified through PCR and cloned into the ampicillin-resistant arabinose-inducible vector pBAD24 (handled with XbaI enzyme) through seamless cloning by ClonExpress Ultra One Step Cloning Kit (Vazyme, China), respectively. The *tmexC3.2-tmexD3.3-toprJ1b* cluster together with its immediately 183-bp upstream region and 145-bp downstream region from strain HD6454 was amplified through PCR and cloned into the ampicillin-resistant vector pUC18 (handled with HindIII and EcoRI enzymes) through seamless cloning by ClonExpress Ultra One Step Cloning Kit (Vazyme, China).
